# The Psychometric Properties of CollaboRATE: A Fast and Frugal Patient-Reported Measure of the Shared Decision-Making Process

**DOI:** 10.2196/jmir.3085

**Published:** 2014-01-03

**Authors:** Paul James Barr, Rachel Thompson, Thom Walsh, Stuart W Grande, Elissa M Ozanne, Glyn Elwyn

**Affiliations:** ^1^The Dartmouth Center for Health Care Delivery ScienceDartmouth CollegeHanover, NHUnited States; ^2^Institute for Health Policy StudiesDepartment of SurgeryUniversity of California, San Francisco,San Francisco, CAUnited States; ^3^The Dartmouth Institute for Health Policy and Clinical PracticeDartmouth CollegeHanover, NHUnited States

**Keywords:** decision making, physician-patient relations, psychometrics/Instrumentation, patient participation, questionnaires, Internet

## Abstract

**Background:**

Patient-centered health care is a central component of current health policy agendas. Shared decision making (SDM) is considered to be the pinnacle of patient engagement and methods to promote this are becoming commonplace. However, the measurement of SDM continues to prove challenging. Reviews have highlighted the need for a patient-reported measure of SDM that is practical, valid, and reliable to assist implementation efforts. In consultation with patients, we developed CollaboRATE, a 3-item measure of the SDM process.

**Objective:**

There is a need for scalable patient-reported measure of the SDM process. In the current project, we assessed the psychometric properties of CollaboRATE.

**Methods:**

A representative sample of the US population were recruited online and were randomly allocated to view 1 of 6 simulated doctor-patient encounters in January 2013. Three dimensions of SDM were manipulated in the encounters: (1) explanation of the health issue, (2) elicitation of patient preferences, and (3) integration of patient preferences. Participants then completed CollaboRATE (possible scores 0-100) in addition to 2 other patient-reported measures of SDM: the 9-item Shared Decision Decision Making Questionnaire (SDM-Q-9) and the Doctor Facilitation subscale of the Patient’s Perceived Involvement in Care Scale (PICS). A subsample of participants was resurveyed between 7 and 14 days after the initial survey. We assessed CollaboRATE’s discriminative, concurrent, and divergent validity, intrarater reliability, and sensitivity to change.

**Results:**

The final sample consisted of 1341 participants. CollaboRATE demonstrated discriminative validity, with a significant increase in CollaboRATE score as the number of core dimensions of SDM increased from zero (mean score: 46.0, 95% CI 42.4-49.6) to 3 (mean score 85.8, 95% CI 83.2-88.4). CollaboRATE also demonstrated concurrent validity with other measures of SDM, excellent intrarater reliability, and sensitivity to change; however, divergent validity was not demonstrated.

**Conclusions:**

The fast and frugal nature of CollaboRATE lends itself to routine clinical use. Further assessment of CollaboRATE in real-world settings is required.

## Introduction

Health care that is patient-centered and supports patient engagement has become an integral aspect of health policy [1-3]. Shared decision making (SDM) has been described as the pinnacle of patient-centered care [4], relevant to managing long-term conditions and situations where multiple treatment options exist. However, to date, implementation has been limited [2,5]. To encourage adoption, SDM has been included in the Patient Protection and Affordable Care Act, as a quality metric for new health care payment and service delivery models [2]. The challenge of developing a measure of the SDM process that is psychometrically sound and suitable for use in routine care forms a barrier to the realization of this plan [6,7] and impedes SDM implementation [8,9].

Measuring the SDM process using observational instruments is laborious, costly, and not conducive to rapid data feedback. Patient-reported measurement of the SDM process may be implemented more successfully. We found 5 such measures: the dyadic OPTION scale [10], the Facilitation of Patient Involvement in Care Scale [11], the Perceived Involvement in Care Scale (PICS) [12], the 9-item Shared Decision Making Questionnaire (SDM-Q-9) [13], and the modified Control Preferences Scale [14]. Four of these measures [10-13] contain 5 or more items, which introduces a patient burden that complicates their integration into usual care. All 5 measures also refer explicitly to a “decision” despite recognition that patients may not always realize that a decision has been made [15,16]. Three of the measures [10,12,13] refer to a single decision, limiting their applicability for health care encounters in which several decisions are made [17,18] and although the psychometric properties of some measures are promising [7], important qualities, such as discriminative validity and intrarater reliability, are often unreported.

A fast and frugal, valid and reliable, patient-reported measure of the SDM process that is applicable to a wide range of clinical settings—especially the primary care setting where varied and often unanticipated decisions are made—is needed. Encouraged by the success of short health measures in other fields [19-23], we developed a 3-item measure of the SDM process, CollaboRATE, in partnership with patients [24,25]. CollaboRATE represents a formative measurement model, assessing the extent to which each of 3 core shared decision-making tasks (or dimensions) are present in a clinical encounter: (1) explanation of the health issue, (2) elicitation of patient preferences, and (3) integration of patient preferences [24]. To date, we have completed the first of 3 planned stages in the development of CollaboRATE: (1) item development with target users, (2) psychometric performance in simulated encounters, and (3) psychometric properties in real clinical populations. In the first stage, we conducted a series of cognitive interviews, where we have shown CollaboRATE to be fast to complete, easy to understand, and to consist of items that are interpreted in the way intended [24]. Our aim in this study, the second stage of CollaboRATE development, was to assess the psychometric properties of CollaboRATE using simulated clinical encounters.

## Methods

### Participants

Participants were adults, 18 years of age or older, residing in the United States, and proficient in English. CollaboRATE was designed to be used in any health care encounter. As such, the target population for CollaboRATE is any person visiting a health provider. Therefore, recruitment quotas, based on the 2010 US Census, were imposed to ensure the sample approximated the US population in terms of gender, age, and educational attainment. Participants were recruited via Survey Sampling International (Shelton, CT), an online survey sampling company with experience in sampling participants for health care research. Survey Sampling International provides small incentives for participation; all respondents were entered into a quarterly draw for US $12,500. The Internet is now a well-established and recognized mode of recruiting participants into research allowing investigators to include hard to reach populations, such as ethnic minorities, with the potential to reduce measurement error, missing data, and respondent attrition. In the recent US Census (2011), 71.7% of Americans reported having access to the Internet at home [26]. The representativeness of data gathered from Internet panels has been shown to be comparable to that from probability-based general population samples [27].

### Simulated Encounters

**Figure 1:**
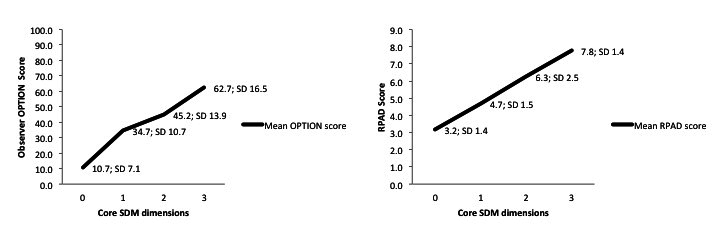
Independent observer ratings (n=7) of the simulated clinical encounters using the observer OPTION Scale and Rochester Participatory Decision-Making Scale.

We created a series of simulated encounters using avatars with audio overlay, where a female patient consulted a male clinician about a prolapsed lumbar disk. Each encounter included zero, 1, 2, or 3 dimensions of SDM (Table 1). In total, 6 encounters were created. No encounters were created that included preference integration in the absence of preference elicitation because this was considered implausible. Encounters were scripted to represent realistic encounters, were spoken by volunteers with American accents, and overlaid on computer animations (Multimedia Appendices 1-6). Seven trained independent raters assessed the level of SDM in each encounter using 2 validated observational measures: the Observer OPTION measure [28] and the Rochester Participatory Decision-Making Scale (RPAD) [29]. As expected, observer ratings demonstrated a linear increase in the mean level of SDM as the number of dimensions increased (Figure 1).

**Table 1:** Number of core dimensions of shared decision making (SDM) included in each simulated encounter.

Encounter	Level of SDM	Dimensions of SDM	Explanation^a^	Preference elicitation^b^	Preference integration^c^	Length (min:s)
1	None	0	No	No	n/a	2:10
2	Low	1	Yes	No	n/a	6:05
3	Low	1	No	Yes	No	3:55
4	Medium	2	Yes	Yes	No	7:52
5	Medium	2	No	Yes	Yes	4:49
6	High	3	Yes	Yes	Yes	8:45

^a^Thorough explanation of health-related information to patient.

^b^Patients’ health-related preferences, views, or opinions elicited.

^c^Patients’ preferences integrated in decision making.

### Measures

#### CollaboRATE

CollaboRATE is a 3-item measure of the SDM process. Items included are:

1. How much effort was made to help you understand your health issues?
2. How much effort was made to listen to the things that matter most to you about your health issues?
3. How much effort was made to include what matters most to you in choosing what to do next?

Participants are instructed to reflect on a health care encounter and then asked to complete the CollaboRATE survey. We administered 2 different response scales to examine their psychometric properties separately. CollaboRATE-10 was a 10-point anchored scale, ranging from 0 (no effort was made) to 9 (every effort was made). CollaboRATE-5 was a 5-point Likert scale, with responses of 0 (no effort was made), 1 (a little effort was made), 2 (some effort was made), 3 (a lot of effort was made), and 4 (every effort was made). We also used 2 scoring methods to enable us to examine their psychometric properties separately. For CollaboRATE mean, we summed participant’s scores on the 3 items and multiplied by 3.704, transforming to a scale from 0 to 100 (for CollaboRATE-10) and the sum of participant’s scores on the 3 items on the original scale from 0 to 12 (for CollaboRATE-5). For CollaboRATE top score, we coded participants as 1 (yes) when they recorded the highest response on the scale for all 3 items and as 0 (no) in all other situations.

#### Other Patient-Reported Measures of the Shared Decision-Making Process

We administered the 9-item SDM-Q-9 [13]. Responses were on a 6-point Likert scale ranging from completely disagree to completely agree with total scores on the survey ranging from 0 to 100. We also administered the 5-item Doctor Facilitation subscale of the PICS (PICS-DFS) [12]. Responses were on binary scale (yes or no) and total scores on the survey ranged from 0 to 5.

#### Clinician Technical Skills

We asked, “How would you rate the technical skills (thoroughness, carefulness, competence) of the provider in the video?” [30] to measure clinician technical skills. Responses were coded as 1 (excellent) or 0 (very good, good, fair, or poor).

#### Participant Characteristics

We assessed participants’ gender, age, educational attainment [31], ethnicity and race [32], and language(s) spoken at home [33] using standard measures. We assessed health care utilization, measured using a single item, “In the last 12 months, did you make any appointment to see a specialist” with a yes/no response option [34]. Self-reported health status was measured using 2 questions: “Do you have any long-standing illness or disability?” (yes/no response option) and, if yes, “Does this illness or disability limit your activities in any way?” (yes/no response option) [35]. Decision-making role preferences were measured using the Control Preferences Scale [36].

### Procedure

Prospective participants were provided with a link to an online information sheet. On the information sheet, participants were informed of the purpose of the survey, the time needed to complete the survey (approximately 15 minutes), and ensured that all data would be stored securely, confidentially, and used only for the purpose of the research project. They were given the number and email of a member of the study team (PJB) to contact if they had any questions. Those who consented were able to enter the online survey system. The survey was created by the research team, piloted with academics and members of the public (n=10) to refine wording, and hosted in Qualtrics, a company specializing in online survey design and data capture. Participants completed items assessing their characteristics and then were randomly allocated by a survey software algorithm to view one of the simulated encounters. Participants were restricted to viewing the simulated encounter once. Potential participants were eligible for inclusion only if they viewed the whole encounter, took the minimum amount of time required to complete the survey questions (3 minutes for the initial survey and 45 seconds for the resurvey), and completed the survey within 1 hour of commencement. Participants were asked to imagine themselves in the position of the patient and then to assess the encounter by completing CollaboRATE, SDM-Q-9, and PICS-DFS. CollaboRATE was administered using both response scales; the presentation of response scales was counterbalanced to attenuate possible order effects. Participants were prevented from making multiple survey entries. This was achieved by preventing respondents with the same Internet Protocol (IP) address from taking the survey again.

A random subsample of participants from each of the encounters was resurveyed between 1 and 2 weeks after initial survey completion [37]. Some of these participants were shown the same encounter a second time, whereas others were shown a different encounter. Participants were asked to complete CollaboRATE using both sets of response scales, which were again counterbalanced. The Committee for the Protection of Human Subjects at the Dartmouth College Institutional Review Board (IRB) approved the study (CPHS #23687).

### Statistical Analysis

The statistical analyses conducted to assess the psychometric properties of CollaboRATE are provided in Table 2. We conducted all analyses using both response scales of CollaboRATE (CollaboRATE-10 and CollaboRATE-5), and both scoring methods (CollaboRATE mean and CollaboRATE top score). As CollaboRATE represents a formative model of SDM, we did not assess internal consistency. Similarly, we did not assess floor or ceiling effects as because the artificial manipulation makes such an assessment invalid. Analyses were conducted using Stata 12 (StataCorp LP, College Station, TX, USA).

**Table 2:** Statistical analyses conducted to assess psychometric properties of CollaboRATE.

Psychometric property	Definition	Assessment^a^	CollaboRATE analyses	
			Mean	Top score
Discriminative validity	Ability of the measure to yield low scores when the construct under measurement is absent, and higher scores as the presence of the construct increases [37]	Between-dimension comparisons of CollaboRATE scores	ANOVA, planned comparisons (between-groups *t* test or Welch test)	Chi-square test
Concurrent validity	Presence of correlation between measures that claim to measure the same construct [37]	Relationship between CollaboRATE and the 2 other measures of SDM (SDM-Q-9 and PICS-DFS)	Pearson product moment correlation (*r*) [38]	Point-biserial correlation (rpb) [39]
Divergent validity	Absence of correlation between measures that claim to measure different constructs [37]	Relationship between CollaboRATE and the clinician technical skills question	Pearson product moment correlation (*r*)	Point-biserial correlation (rpb)
Intrarater reliability	Consistency of ratings of the same encounter, across 2 time points by the same rater [37]	Comparison of CollaboRATE scores on initial survey and resurvey for participants exposed to the same encounter	Intraclass correlation coefficients (ICC 2,2; 2-way mixed effects model of absolute agreement)	Cohen’s kappa coefficient [40]
Sensitivity to change	Ability of the measure to detect change in the specified construct, regardless of whether it is deemed meaningful to the decision maker [37,41]	Comparison of CollaboRATE scores on initial survey and resurvey for participants exposed to the “opposite” encounter on resurvey (e.g., low SDM on initial survey, high SDM on resurvey)	Paired *t* test	McNemar’s test

^a^SDM: shared decision making; SDM-Q-9: 9-item Shared Decision Making Questionnaire; PICS-DFS: 5-item Doctor Facilitation subscale of the Perceived Involvement in Care Scale.

### Sample Size Calculation

To detect an estimated 15% difference in the proportion of participants with a top score on CollaboRATE between the encounter with 3 dimensions present (estimated 75% top score) and an encounter with 2 dimensions present (estimated 60% top score), with 90% power, 216 participants per encounter were required. We planned to resurvey 30 participants initially exposed to each of the encounters who would be exposed to the same encounter again (providing 95% power to detect a minimum intraclass correlation coefficient, ICC, of 0.65) and a further 30 participants initially exposed to the 2 extreme encounters (zero dimensions and 3 dimensions) who would be exposed to the opposite encounter.

## Results

### Participant Flow

A total of 2026 participants completed the initial survey. Before analysis, 685 (33.8%) were excluded for taking less than the minimum reasonable time to complete the survey, resulting in a total of 1341 eligible participants included. A total of 388 participants were approached for resurvey. Prior to analysis, 137 (35.3%) were excluded for taking less than the minimum reasonable time to complete the survey, resulting in a total of 251 eligible participants included in the resurvey.

### Participant Characteristics

Characteristics of the participants were similar to that of the US population. Participants’ characteristics across the dimensions were comparable (Table 3) although there were statistically significant differences in race (*P*=.04). 6.94% (93/1341) of participants did not report age or gender, but no differences in CollaboRATE scores were found between those that did and did not report age (*P*=.45) or gender (*P*=.76). The acceptability of CollaboRATE items was demonstrated by less than 1% (8/1341) of participants missing any of the items.

### Discriminative Validity

The discriminative validity of CollaboRATE was demonstrated with significant increases in scores as progressively more of the dimensions were included in the encounters (Table 4). For all analyses, a significant overall association between CollaboRATE and number of dimensions was found (data available on request).

This was true for both response scales and both scoring methods. The discriminative validity of the 2 other measures of SDM was also demonstrated. No significant differences were observed in CollaboRATE between the 2 encounters that included 1 dimension of SDM, nor between the 2 encounters that included 2 dimensions of SDM, on either response scales or scoring method (analysis available upon request). A further description of CollaboRATE scores per item is presented in Table 5.

**Table 3:** Participant characteristics by group.

Sociodemographic and health care characteristics			Number of dimensions^a^				Total (n=1341)	US population^b^
			0 (n=270)	1 (n=443)	2 (n=425)	3 (n=203)		
**Gender, n (%)**								
	Female		128 (50.0)	218 (53)	223 (57.2)	104 (54.5)	673 (53.9)	50.8%
	Male		128 (50.0)	193 (47.0)	167 (42.8)	87 (45.6)	575 (46.1)	49.2%
**Age (years), n (%)**								
	18-44		85 (39.5)	172 (50.3)	165 (50.5)	77 (46.7)	499 (47.6)	48.1%
	45-64		79 (36.7)	110 (32.2)	102 (31.2)	55 (30.3)	346 (33.0)	34.7%
	65+		51 (23.7)	60 (17.5)	60 (18.4)	33 (20.0)	204 (19.5)	17.2%
**Educational attainment**								
	High school graduate or less		108 (40.3)	179 (40.4)	169 (40.0)	84 (41.6)	540 (40.4)	42.7%
	Some college, no degree		56 (20.9)	103 (23.3)	100 (23.6)	41 (20.3)	300 (22.5)	16.7%
	Associate’s or bachelor’s degree		79 (29.5)	120 (27.1)	126 (29.8)	55 (27.2)	380 (28.4)	29.5%
	Master’s, professional, or doctoral degree		25 (9.3)	41 (9.3)	28 (6.6)	22 (10.9)	116 (8.7)	11.1%
**Ethnicity**								
	Hispanic or Latino		21 (8.1)	28 (6.4)	32 (7.7)	17 (8.6)	98 (7.5)	16.3%
	Not Hispanic or Latino		238 (91.9)	409 (93.6)	384 (92.3)	181 (91.4)	1212 (92.5)	83.7%
	White alone		205 (86.1)	315 (77.0)	314 (81.8)	148 (81.8)	982 (81.0)	63.7%
**Race**								
	**One race**		260 (97)	423 (96.1)	413 (97.6)	198 (98.5)	1299 (97.5)	97.1%
		White	229 (85.5)	341 (77.5)	342 (80.9)	161 (80.1)	1073 (80.6)	72.4%
		Black or African American	18 (6.7)	45 (10.2)	39 (9.2)	27 (13.4)	129 (9.7)	12.6%
		American Indian and Alaska Native	4 (1.5)	4 (0.9)	6 (1.4)	0	14 (1.1)	0.9%
		Asian	4 (1.5)	21 (4.8)	15 (3.6)	4 (2.0)	44 (3.3)	4.8%
		Native Hawaiian and Other Pacific Islander	0	4 (0.9)	0	0	4 (0.3)	0.2%
		Some other race	5 (1.9)	13 (3.0)	11 (2.6)	6 (3.0)	35 (2.6)	6.2%
	Two or more races		8 (3.0)	17 (3.9)	10 (2.4)	3 (1.5)	33 (2.5)	2.9%
**Language spoken at home**								
	English only		243 (91.4)	386 (89.4)	370 (88.5)	87 (42.9)	1177 (89.9)	80.4%
	Language other than English		23 (8.7)	46 (10.7)	48 (11.5)	116 (57.1)	133 (10.2)	19.6%
**Health care experiences and preferences**								
	**Long-standing illness or disability**							
		Yes, and limits activities	55 (20.6)	117 (26.6)	122 (28.8)	62 (30.8)	356 (26.7)	–
		Yes, and does not limit activities	26 (9.7)	38 (8.6)	37 (8.7)	23 (11.4)	124 (9.3)	–
		No	186 (68.7)	285 (64.8)	264 (62.4)	116 (57.7)	851 (63.9)	–
	**Specialist appointment in last 12 months**							
		Yes	120 (44.6)	228 (51.6)	223 (52.5)	104 (51.2)	661 (49.4)	–
		No	149 (55.4)	214 (48.4)	202 (47.5)	99 (48.8)	678 (50.6)	–
	**Decision-making role preferences**							
		Patient alone	37 (13.8)	74 (16.7)	64 (15.1)	35 (17.2)	210 (15.7)	–
		Patient with provider input	117 (43.5)	167 (37.7)	159 (37.4)	69 (34.0)	512 (38.2)	–
		Shared	84 (31.2)	162 (36.6)	164 (38.6)	84 (41.4)	494 (36.9)	–
		Provider with patient input	17 (6.3)	22 (5.0)	17 (4.0)	11 (5.4)	67 (5.0)	–
		Provider alone	14 (5.2)	18 (4.1)	21 (4.9)	4 (2.0)	57 (4.2)	–

^a^Frequencies may not sum to the total due to missing data.

^b^Gender and age data were taken from the 2010 Census [42], educational attainment data correspond to the population aged ≥25 years and were taken from the Current Population Survey 2012 Annual Social and Economic Supplement [31], ethnicity and race data were taken from 2010 Census [43], and language data were taken from the 2006-2008 American Community Survey [33].

**Table 4:** Discriminative validity of CollaboRATE, the 9-item Shared Decision Making Questionnaire (SDM-Q-9), and the 5-item Doctor Facilitation subscale of the Perceived Involvement in Care Scale (PICS-DFS).

Discriminative validity	Number of dimensions				Contrasts between dimensions^a^										Valid^b^
	0 (n=270)	1 (n=443)	2 (n=425)	3 (n=203)	0 vs 1			1 vs 2			2 vs 3				
					*t* (*df*)	χ^2^_1_	*P*	*t* (*df*)	χ^2^_1_	*P*	*t* (*df*)		χ^2^_1_	*P*	
CollaboRATE-10, mean (SD)	46.0 (29.9)	69.6 (26.2)	82.0 (21.6)	85.8 (19.1)	–10.68 (505.1)		<.001	–7.58 (844.1)		<.001	–2.25 (447.7)			.01	Yes
CollaboRATE-5, mean (SD)	5.2 (3.4)	7.8 (3.1)	9.4 (2.6)	10.0 (2.3)	–10.15 (525.2)		<.001	–8.37 (845.0)		<.001	–2.66 (435.3)			.008	Yes
CollaboRATE-10 top score, n (%)	13 (4.9)	79 (17.9)	131 (31.1)	81 (39.9)		24.9	<.001		20.5	<.001			4.7	.03	Yes
CollaboRATE-5 top score, n (%)	16 (6.0)	76 (17.2)	136 (32.2)	85 (42.3)		18.4	<.001		26.0	<.001			6.1	.01	Yes
SDM-Q-9, mean (SD)	37.1 (27.9)	63.2 (23.4)	75.1 (19.8)	82.0 (16.0)	–12.85 (490.6)		<.001	–8.09 (852.9)		<.001	–4.69 (484.6)			<.001	Yes
PICS-DFS, mean (SD)	1.60 (1.9)	3.2 (1.7)	3.9 (1.2)	4.3 (0.9)	–11.64 (510.8)		<.001	–7.07 (798.2)		<.001	–4.64 (496.4)			<.001	Yes

^a^Two-sample *t* test with unequal variances for contrasts of means.

^b^Yes=psychometric property found in this sample; no=psychometric property not found in this sample.

**Table 5:** CollaboRATE scores by item.

CollaboRATE items		Number of dimensions			
		0 (n=270)	1 (n=443)	2 (n=425)	3 (n=203)
**CollaboRATE-10, mean (SD)**					
	Item 1 (information)	4.50 (2.76)	6.63 (2.36)	7.48 (1.94)	7.70 (1.86)
	Item 2 (preference)	4.19 (2.83)	6.25 (2.49)	7.36 (2.07)	7.69 (1.75)
	Item 3 (integration)	3.77 (2.96)	5.94 (2.74)	7.32 (2.16)	7.80 (1.73)
**CollaboRATE-5, mean (SD)**					
	Item 1 (information)	1.98 (1.10)	2.74 (1.02)	3.18 (0.85)	3.34 (0.84)
	Item 2 (preference)	1.73 (1.21)	2.58 (1.31)	3.14 (0.95)	3.27 (0.83)
	Item 3 (integration)	1.51 (1.33)	2.49 (1.25)	3.13 (1.02)	3.39 (0.82)
**CollaboRATE-10 top score, n (%)**					
	Item 1 (information)	22 (8.2)	117 (26.5)	175 (41.4)	97 (47.8)
	Item 2 (preference)	20 (7.5)	103 (23.4)	172 (40.6)	95 (46.8)
	Item 3 (integration)	23 (8.6)	98 (22.1)	180 (42.6)	99 (48.8)
**CollaboRATE-5 top score, n (%)**					
	Item 1 (information)	26 (9.7)	109 (24.6)	175 (41.3)	104 (51.5)
	Item 2 (preference)	24 (8.9)	111 (25.2)	184 (43.3)	95 (47.0)
	Item 3 (integration)	24 (9.0)	111 (25.1)	194 (45.8)	112 (55.2)

### Concurrent Validity, Divergent Validity, and Intrarater Reliability

The concurrent validity of CollaboRATE was demonstrated with moderate to strong positive correlations between the 2 other measures of SDM for both response scales and both scoring methods (see Table 6). Divergent validity of CollaboRATE was not demonstrated, with moderate to strong positive correlations also observed with the clinician technical skills rating for both response scales and both scoring methods. Intrarater reliability of CollaboRATE mean scores was demonstrated for both response scales, with excellent intraclass correlations observed between Time 1, initial survey completion, and Time 2, resurvey, scores. Intrarater reliability of CollaboRATE top scores was also demonstrated for both response scales, with moderate agreement observed between Time 1 and Time 2 scores (Table 6).

### Sensitivity to Change

Sensitivity to change of CollaboRATE was demonstrated with significant differences observed between scores for encounters with zero and 3 dimensions of SDM (within participants) for both response scales and both scoring methods (Table 7).

**Table 6:** Concurrent validity, divergent validity, and intrarater reliability of CollaboRATE.

Psychometric properties of CollaboRATE		Statistic	95% CI	*P*	Relationship	Valid/reliable^a^
**Concurrent validity (with SDM-Q-9)**						
	CollaboRATE-10 mean	*r*=0.79	0.77, 0.81	<.001	Strong, positive	Yes
	CollaboRATE-5 mean	*r*=0.80	0.78, 0.82	<.001	Strong, positive	Yes
	CollaboRATE-10 top score	rpb=0.49	0.45, 0.53	<.001	Moderate, positive	Yes
	CollaboRATE-5 top score	rpb=0.50	0.46, 0.54	<.001	Strong, positive	Yes
**Concurrent validity (with PICS-DFS)**						
	CollaboRATE-10 mean	*r*=0.67	0.64, 0.70	<.001	Strong, positive	Yes
	CollaboRATE-5 mean	*r*=0.68	0.65, 0.71	<.001	Strong, positive	Yes
	CollaboRATE-10 top score	rpb=0.36	0.31, 0.41	<.001	Moderate, positive	Yes
	CollaboRATE-5 top score	rpb=0.37	0.32, 0.42	<.001	Moderate, positive	Yes
**Divergent validity (with clinician technical skills rating)**						
	CollaboRATE-10 mean	rpb=0.42	0.37, 0.46	<.001	Moderate, positive	No
	CollaboRATE-5 mean	rpb=0.46	0.42, 0.51	<.001	Moderate, positive	No
	CollaboRATE-10 top score	Agreement=83.4 % Kappa= 0.53	0.48, 0.59	<.001	Moderate	No
	CollaboRATE-5 top score	Agreement=83.8 % kappa=0.55	0.50, 0.60	<.001	Moderate	No
**Intrarater reliability (Time 1 to Time 2)**						
	CollaboRATE-10 mean	ICC (2,2)=0.86	0.82, 0.90	<.001	Excellent	Yes
	CollaboRATE-5 mean	ICC (2,2)=0.82	0.76, 0.87	<.001	Excellent	Yes
	CollaboRATE-10 top score	Agreement=84.7 % kappa=0.56	0.42, 0.70	<.001	Moderate	Yes
	CollaboRATE-5 top score	Agreement=82.4 % kappa=0.58	0.44, 0.72	<.001	Moderate	Yes

^a^Yes=psychometric property found in this sample; no=psychometric property not found in this sample.

**Table 7:** Sensitivity to change of CollaboRATE.

CollaboRATE	Time 1 to Time 2 (n=29)					Time 1 to Time 2 (n=33)				
	Dimensions		Statistic			Dimensions		Statistic		
	0	3	*t*_28_	χ^2^_1_	*P*	3	0	*t* (*df*)	χ^2^_1_	*P*
CollaboRATE-10 mean, mean (SD)	38.0 (29.0)	78.9 (28.0)	–6.75		<.001	82.2 (18.3)	66.3 (25.5)	3.58 (32)		<.001
CollaboRATE-5 mean,^a^ mean (SD)	4.5 (3.2)	9.0 (3.8)	–5.87		<.001	9.7 (2.6)	7.4 (3.2)	4.73 (31)		<.001
CollaboRATE-10 top score, n (%)	1 (3.5)	12 (41.4)		11.0	.001	11 (33.3)	3 (9.1)		8.0	.008
CollaboRATE-5 top score, n (%)	1 (3.5)	13 (44.8)		12.0	<.001	13 (39.4)	5 (15.2)		6.4	.02

^a^1 missing response for CollaboRATE-5 Time 1 (3 dimensions) to Time 2 (0 dimensions).

## Discussion

### Principal Findings

In simulated patient-clinician encounters, CollaboRATE, a patient-reported measure of the SDM process, demonstrated discriminative validity, concurrent validity, intrarater reliability, and sensitivity to change. Divergent validity was not demonstrated. Although further testing in real-world clinical care is needed, these results provide a solid foundation on which to consider this measure a fast and frugal measure of the SDM process.

CollaboRATE discriminated between all levels of SDM. It was particularly effective when discriminating between the absence and presence of any level of SDM. Although the discriminative ability of CollaboRATE was evident between moderate and high SDM encounters, the magnitude of differences was smaller. A greater number of recordings would be required to detect differences between moderate and high SDM in real-world settings.

CollaboRATE performed as well as the 2 most-commonly used patient-reported measures of SDM process. All 3 measures (CollaboRATE, SDM-Q-9, and the PICS-DFS) demonstrated excellent psychometric qualities, including discriminative validity (previously unreported for both SDM-Q-9 and the PICS-DFS). CollaboRATE scores remained consistent when retested over a 1- to 2-week period. CollaboRATE was also capable of detecting a change on resurvey in the level of SDM when participants viewed a clinical encounter with a different number of core dimensions.

There was little difference in the psychometric properties of CollaboRATE when a 10-point anchored scale or 5-point Likert response scale was used. Further discussion and testing with patients and clinicians, in real clinics is required to decide which is preferred. In addition, top score analysis was also conducted as part of our analysis and mirrored the psychometric properties of CollaboRATE when treated as a continuous outcome, with the exception of reduced intrarater reliability.

### Strengths and Limitations of the Study Method

A strength of our method is that the use of simulated encounters, delivered via the Internet, allowed us to examine discriminative validity in ways that are not possible in clinical settings. The use of simulated medical encounters in this manner has been used successfully in previous studies and is deemed an important intermediary step to real-world testing [10,44-46]. It could be argued that assessing a measure under idealized circumstances is highly desirable because it is less time consuming, less intrusive for participants, and less costly. Moreover, if a measure cannot perform under ideal and controlled circumstances, it is unlikely to succeed in the mire of clinical practice. In addition, we have successfully demonstrated that the Internet can be used to successfully deliver and conduct this type of psychometric assessment in the field of SDM. We hope to encourage other measure development researchers to consider this approach in the future before testing in the clinical setting. Our choice of animated characters rather than real-life video recordings was to avoid potential rater bias that has been commonly reported with the latter [47]. The simulated encounters are freely available for use as teaching or research resources (Multimedia Appendices 1-6).

A limitation is potential confounding because of the differing durations of the simulated encounters, which increased as more dimensions of SDM were included. However, we argue that this is also likely reflective of how SDM might increase in the clinical setting. In addition, the varying length of times across each of the 6 scenarios could not be standardized without introducing more bias. We plan to assess the impact of consultation length on SDM in usual care. We were also limited to creating encounters that dealt with only 1 health issue. This may be reflective of specialist care, but it is less reflective of primary care. Our choice of clinician technical skills as measure of divergent validity appeared inadequate as none of the measures could meet this criteria using this question in the current sample. Finally, there was the potential of introducing selection bias, as approximately 35% of participants were excluded for not taking the minimal required time to view the encounter and complete the survey. However, we feel this risk was low as are use of quotas ensured a representative sample of the US population.

### Results in Context

Our findings contribute further evidence that short patient-reported measures can produce valid and reliable results [19,22,48,49] and we believe that CollaboRATE addresses this gap in the field of SDM process measurement [7]. The psychometric qualities of CollaboRATE in the current study compare well with the reported psychometric properties of existing measures [6,7]. We demonstrated intrarater reliability, as has been shown for the Facilitation of Patient Involvement in Care Scale [11]. We also demonstrated CollaboRATE’s discriminative validity and sensitivity to change. To our knowledge, this is the first time these aspects of validity have been demonstrated in a measure of the SDM process. Although short whole-encounter measures of the SDM process exist currently [11,14], we believe CollaboRATE is more understandable for patients because it avoids explicit reference to decisions made within the encounter [15,16].

### Implications

To date, measures of the SDM process are not routinely implemented in clinical practice. We believe that CollaboRATE can assist in this effort because it is easy to understand and allows for uncomplicated analysis. Our previous work demonstrates that CollaboRATE is also easily administered to patients and has high face validity [24]. In addition there are practical benefits of short tools for both research (eg, reduced respondent burden), and policy (eg, ease of interpretation, implementation, and cost) [20,21]. The potential of CollaboRATE to assess SDM generically increases the potential scope of its use, whether patients seek help for long-term conditions or in situations where alternative treatments need to be compared. Although lengthier observer- and patient-reported measures of the SDM process can provide more detail about the consultation, CollaboRATE is better positioned to be used on a larger scale to produce valid and reliable measurement of the SDM process while also enabling faster feedback to clinics and clinicians. This patient-reported feedback can have positive effects on clinical practice [50] and patient participation in medical care associated with a range of positive health outcomes [51].

### Conclusion

We have developed a fast and frugal measure of the SDM process that has sound psychometric properties when tested in a simulated setting. Stage 3 evaluation of CollaboRATE in real-world clinical settings, including its psychometric properties and feasibility, is now required.

## Multimedia Appendix 1

Simulated doctor-patient encounter with no attempt at shared decision making.

## Multimedia Appendix 2

Simulated doctor-patient encounter with a low level of shared decision making: detailed Information on health issue provided, no preference elicitation or integration.

## Multimedia Appendix 3

Simulated doctor-patient encounter with a low level of shared decision making: preference elicitation, little information on health issue provided and no preference integration.

## Multimedia Appendix 4

Simulated doctor-patient encounter with a moderate level of shared decision making: detailed Information on health issue provided and preferences elicited, no preference integration.

## Multimedia Appendix 5

Simulated doctor-patient encounter with a moderate level of shared decision making: preferences elicited and integrated, little information on health issue provided.

## Multimedia Appendix 6

Simulated doctor-patient encounter with a high level of shared decision making: detailed Information on health issue provided, preferences elicited and integrated.

## Abbreviations

CPHS: Committee for the Protection of Human Subjects
ICC: intraclass correlation
IP: Internet Protocol
IRB: Institutional Review Board
PICS-DFS: 5-item Doctor Facilitation subscale of the PICS
PICS: Perceived Involvement in Care Scale
RPAD: Rochester Participatory Decision-Making Scale
SDM-Q-9: 9-item Shared Decision Making Questionnaire
SDM: shared decision making
