# Correction: The Psychometric Properties of CollaboRATE: A Fast and Frugal Patient-Reported Measure of the Shared Decision-Making Process

**DOI:** 10.2196/jmir.4272

**Published:** 2015-02-06

**Authors:** Paul James Barr, Rachel Thompson, Thom Walsh, Stuart W Grande, Elissa M Ozanne, Glyn Elwyn

**Affiliations:** ^1^The Dartmouth Center for Health Care Delivery ScienceDartmouth CollegeHanover, NHUnited States; ^2^The Institute for Health Policy StudiesDepartment of SurgeryUniversity of California, San FranciscoSan Francisco, CAUnited States; ^3^The Dartmouth Institute for Health Policy and Clinical PracticeDartmouth CollegeHanover, NHUnited States

The authors of “The Psychometric Properties of CollaboRATE: A Fast and Frugal Patient-Reported Measure of the Shared Decision-Making Process” (http://www.jmir.org/2014/1/e2/) have overlooked an error in the Methods section during the submission process. The sentence “We administered 2 different response scales to examine their psychometric properties separately. CollaboRATE-10 was a 10-point anchored scale, ranging from 0 (no effort at all)” should have been “...ranging from 0 (no effort was made)”. This change is also needed in: “CollaboRATE-5 was a 5-point Likert scale, with responses of 0 (no effort at all),” to read “...with responses of 0 (no effort was made)”. These errors have been corrected in the online version of the paper on the JMIR website on February 6, 2015, together with publishing this correction notice. A correction notice has been sent to PubMed and the correct full-text has been resubmitted to Pubmed Central and other full-text repositories.

